# Myristoylation: An Important Protein Modification in the Immune Response

**DOI:** 10.3389/fimmu.2017.00751

**Published:** 2017-06-30

**Authors:** Daniel Ikenna Udenwobele, Ruey-Chyi Su, Sara V. Good, Terry Blake Ball, Shailly Varma Shrivastav, Anuraag Shrivastav

**Affiliations:** ^1^Department of Biology, University of Winnipeg, Winnipeg, MB, Canada; ^2^Department of Biochemistry, University of Nigeria, Nsukka, Enugu, Nigeria; ^3^JC Wilt Infectious Diseases Research Institute, National HIV and Retrovirology Laboratory, Public Health Agency of Canada, Winnipeg, MB, Canada; ^4^Department of Medical Microbiology and Infectious Diseases, University of Manitoba, Winnipeg, MB, Canada; ^5^VastCon Inc., Winnipeg, MB, Canada; ^6^Department of Biochemistry and Medical Genetics, University of Manitoba, Winnipeg, MB, Canada

**Keywords:** myristoylation, N-myristoyltransferase, lipid modification, signal transduction, T cells, human immunodeficiency virus

## Abstract

Protein N-myristoylation is a cotranslational lipidic modification specific to the alpha-amino group of an N-terminal glycine residue of many eukaryotic and viral proteins. The ubiquitous eukaryotic enzyme, N-myristoyltransferase, catalyzes the myristoylation process. Precisely, attachment of a myristoyl group increases specific protein–protein interactions leading to subcellular localization of myristoylated proteins with its signaling partners. The birth of the field of myristoylation, a little over three decades ago, has led to the understanding of the significance of protein myristoylation in regulating cellular signaling pathways in several biological processes especially in carcinogenesis and more recently immune function. This review discusses myristoylation as a prerequisite step in initiating many immune cell signaling cascades. In particular, we discuss the hitherto unappreciated implication of myristoylation during myelopoiesis, innate immune response, lymphopoiesis for T cells, and the formation of the immunological synapse. Furthermore, we discuss the role of myristoylation in inducing the virological synapse during human immunodeficiency virus infection as well as its clinical implication. This review aims to summarize existing knowledge in the field and to highlight gaps in our understanding of the role of myristoylation in immune function so as to further investigate into the dynamics of myristoylation-dependent immune regulation.

## Introduction

The premolecular genomic era was characterized by the assumption that genome size (the *C* value) is directly proportional to gene number and organisms with larger genomes should have a concomitantly larger proteome and greater morphological complexity. By 1951, it was demonstrated that genome size is not correlated with any measure of organism complexity, leading to the so-called *C*-value paradox (1). However, during the past few decades, biochemical and genomic data have largely resolved the paradox: first, genome size is broadly correlated with the amount of middle and highly repetitive sequences and second proteomic complexity in humans and other higher vertebrates is achieved by complex transcriptional and posttranslational modifications. In vertebrates, epigenomic control of gene regulation is highly dynamic and a single multiexonic gene may encode multiple transcripts *via* alternative splicing, which can become proteins which undergo further posttranslational modification; the combination of epigenetic regulation and posttranslational modifications greatly increase the responsiveness of the genome to internal and external conditions. In humans, an estimated 20,000 genes produce ~100,000 proteins *via* alternatively splicing and these ~100,000 proteins undergo extensive posttranslational modifications such as the addition of carbohydrates, phosphate, methyl groups, or lipids. Attachment of lipid moieties is important for intracellular trafficking of proteins and for generating the final native structure of a protein (2).

**Figure 1:**
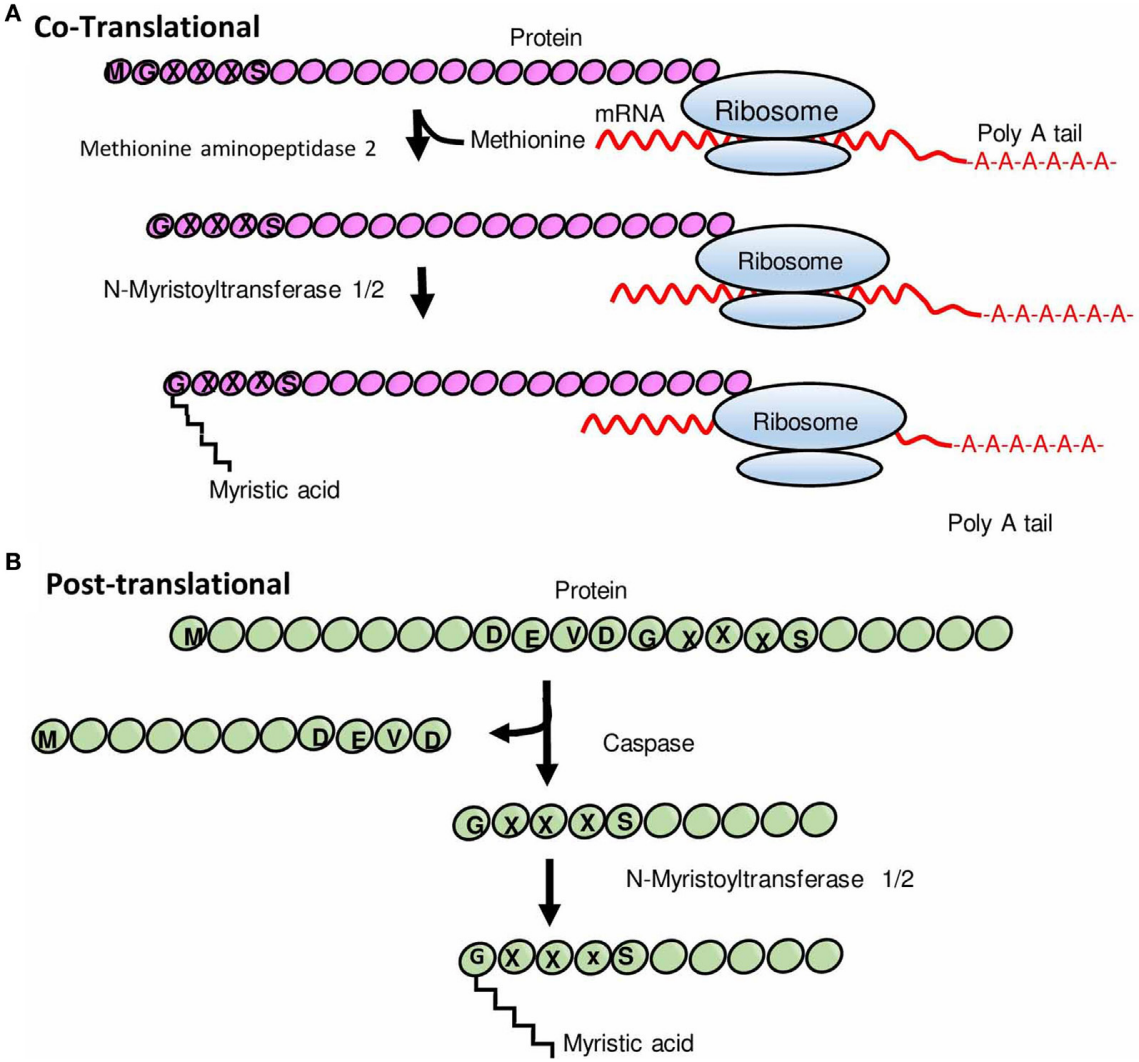
Schematic representation of N-myristoylation of proteins. **(A)** Cotranslational protein N-myristoylation. After the removal of an initiator methionine by methionine aminopeptidase 2, myristic acid (a 14-carbon saturated fatty acid) is transferred to the N-terminal glycine residue of a protein by N-myristoyltransferase (NMT). **(B)** Posttranslational protein N-myristoylation. Some proteins undergo myristoylation posttranslationally wherein proteins are first cleaved by a protease to expose glycine residue and thereafter a myristic acid is covalently attached to glycine residue by NMT. Modified from Ref. (5).

Myristoylation is one such protein lipid modification, which plays vital roles in cellular signaling, protein–protein interaction, and targeting of proteins to endomembrane and plasma membrane systems (3). The attachment of myristic acid to the N-terminus is catalyzed by the ubiquitous eukaryotic enzyme, N-myristoyltransferase (NMT); a prosurvival protein, which uses myristoyl-coenzyme A (CoA) as a substrate (Figure 1). Although myristoylation typically occurs cotranslationally on newly synthesized polypeptides following cleavage of the initiator methionine by methionine aminopeptidase (Figure 1A), there is also evidence that NMT-mediated posttranslational modification of proteins occurs after proteolytic cleavage of an N-terminal glycine residue (Figure 1B) (4).

A fundamental question in the field has been “how does myristoylation regulate protein activity”? The relationship between protein activity, dimensionality, and regulation follows from the PolyA recurrence theorem which stipulates that the probability of a random walk in 1*d* or 2*d* (where *d* = dimensional lattice) returning to its origin is *p* (1) while if *d* > 2, the probability is *p* (0) (6, 7). However, Adam and Delbruck were the first to quantitatively define the dimensionality of a protein. They observed that membrane binding confines a protein to 2*d*, and it is easier for a system in 2*d* to achieve steady state (8–10). Thus, endothelial nitric oxide synthase (eNOS), a myristoylated protein, is confined to the 2*d* of the caveolae where it is more likely to encounter and bind to caveolin than in the 3*d* of the cytoplasm. Also, myristic acid is a hydrophobic moiety, and as the cell microenvironment is hydrophilic, the myristoylated protein is inserted into hydrophobic regions within the cell namely on lipid rafts, the plasma membrane, endoplasmic reticulum (ER), Golgi apparatus, nuclear membrane, and mitochondria. Thus, depending on the subcellular localization of the myristoylated protein, it can regulate diverse cellular functions (11). Myristoylated proteins are adapted to performing biological functions in signal transduction (12, 13), cellular transformation (14), and oncogenesis. Myristic acid is not an abundant fatty acid, it accounts for less than 1% of the total fatty acid in the cell (12) introducing another form of regulation.

Functionally, NMT, enzyme that catalyzes myristoylation, has been found to be critical for cell survival (15–18), regulation of both innate and adaptive immune responses, and human immunodeficiency virus (HIV) infection (19). This review highlights the role of NMT in the early signaling events during the activation of macrophages and T cells, and the regulatory cycle of myristoylated proteins and its clinical importance during HIV replication.

## Protein NMT

The enzyme, myristoyl-CoA:NMT (EC 2.3.1.97), is a member of GCN5-related-N-actyltransferase protein family (17). NMT catalyzes an irreversible acylation process in which a 14-carbon saturated fatty acid, myristic acid, is covalently attached at the N-terminal glycine residue after the removal of initiator methionine by methionine aminopeptidase. In lower eukaryotes, a single gene encodes NMT while in higher eukaryotes two genes, NMT1 and NMT2, encode NMT. Studies on substrate specificity and NMT kinetics were used to design NMT-specific inhibitors which have been demonstrated to have cytotoxic effects (20). Although NMT1 and NMT2 share similar substrate, biochemical and kinetic studies indicate that they have different substrate affinities and are not functionally redundant (21). For instance, Giang and Cravatt reported that while the myristoylation rate of NMT1 is 70% for c-Abl peptide, the myristoylation rate for NMT2 is 50% for the same peptide (22). Ducker et al. demonstrated that NMT isozymes play unique roles in protein myristoylation, apoptosis, and cell cycle by knocking down either NMT1 or NMT2. The study revealed that ablation of NMT1 inhibited cell replication associated with the loss of activation of c-Src and its effector protein focal adhesion kinase (FAK) along with reduction in the signaling through c-Raf/MAP kinase/extrasignal-regulated kinase kinase/extracellular signal-regulated kinase pathway. Depletion of either NMT1 or NMT2 induced apoptosis; NMT2 showed a 2.5-fold greater effect than NMT1. In addition, knockdown of NMT2 expression resulted in the induction of apoptosis by BCL family of proteins. In fact, intramural injection of NMT-specific siRNA, both NMT1 and NMT2 specific siRNAs reduced tumor growth. However, injection of NMT2-specific siRNA alone failed to reduce tumor growth (23). The specificity of NMT substrates has been extensively studied; NMT attaches myristic acid to a consensus amino acid sequence G^1^N^2^X^3^X^4^X^5^X^6^R^7^R^8^ (12). However, many myristoylated proteins beside having Gly at position one of the amino-terminal end were found to have different amino-terminal motifs (12). Johnson and colleagues reported a consensus sequence which in addition to having a Gly residue at the N-terminal ends requires a Ser/Thr residue at the fifth position (24) G^1^X^2^X^3^X^4^S/T^5^X^6^X^7^X^8^. This consensus sequence favors myristoylation and was found in many of the myristoylated proteins. Rudnick et al. (25) reported that myristoylation of proteins occurs *via* ordered sequential bi–bi mechanism, in which all the substrates must bind to the enzyme before the product is released (25). NMT activity regulated phosphorylation and dephosphorylation, which have been shown to be mediated by members of non-receptor tyrosine kinases and Ca^2+^/calmodulin-dependent protein phosphatase, calcineurin (CaN), respectively (17).

## Regulatory Cycle of N-Myristoylated Protein

As alluded earlier, N-myristoylation not only increases protein complexity and function but also adds a certain degree of regulation to myristoylated proteins. N-Myristoylated proteins are directed to the plasma membrane based on the orientation of the myristoyl moiety. The myristoyl group; in a few cases, is sequestered within the hydrophobic pockets and more often than not is exposed on the protein surfaces. Conformational alterations in such proteins modulate myristoyl group’s spatial arrangement resulting in altered hydrophobicity of the proteins. For instance, the myristoyl group in recoverin, which is a calcium sensor protein involved in transduction of light, acts as a myristoyl-Ca^2+^ switch. Recoverin binds Ca^2+^ cooperatively, which leads to the extrusion of the myristoyl group enabling the binding of recoverin to the rod membrane, while in the absence of Ca^2+^, the myristoyl moiety is sequestered within hydrophobic pockets (9, 26, 27). Similarly, exchange of GDP to GTP by guanine nucleotide exchange factor induces a myristoyl-conformational switch that favors membrane targeting of Ras-related GTPases (28). Myristoylation is an irreversible stable modification and as expected the half-life of a myristoylated protein is parallel to that of the nascent polypeptide chain (29). This has sparked debates about how myristoylated proteins are processed after biological events and whether demyristoylation could be a possible mechanism of regulating myristoylated proteins. In one report, macrophage lysate was shown to contain a protease that cleaves myristoylated alanine-rich C kinase substrate (MARCKS) and the cleavage is dependent on myristoylation suggesting that myristoylation is part of the recognition motif for protease activity (30). There are also a few reports suggesting demyristoylation of myristoylated proteins. Demyristoylation activity was reported in a cytoplasmic extract of brain synaptosomes, and demyristoylation was shown to be ATP dependent (31, 32). In a separate report (33), a pool of non-myristoylated MARCKS was isolated from bovine brain suggesting the presence of demyristoylase enzyme at least in neuronal cells, although it could be argued that the presence of non-myristoylated MARCKS was due to protease action (30). Another report suggests that demyristoylation is dependent on the availability of CoA; such that when the concentration of CoA is high, myristoylation is favored while the reverse is true at low concentrations (34). Together, the evidence supporting demyristoylation is still scarce. Further work is required to better understand how myristoylated proteins are regulated during cellular homeostasis.

## The Role of N-Myristoylation in Hematopoiesis

Accumulating evidence demonstrates that myristoylation is an evolutionary conserved lipid modification that plays an important role in cell viability (5, 35–37). A wide array of eukaryotic, viral, and also a few plant proteins have been shown to undergo myristoylation [for review, see Ref. (5, 12, 15, 38)]. Duronio et al. (36) demonstrated that a point mutation in the gene that governs myristoylation *via* homologous recombination leads to recessive lethality in yeast cells. This finding led to several studies that sought to characterize the functional role of myristoylation in signal transduction, since many proteins involved in cellular signaling are myristoylated [see Ref. (5, 23, 35) for review]. The results of these studies are consistent with the biological role of myristoylation in maintaining the viability of organism and cells (39). Earlier, we reported that intercrosses of *NMT1^+/−^* offspring did not produce viable *NMT1^−/−^* offspring and that NMT2 was unable to maintain viability and normal phenotype. These findings led us to conclude that the two isoforms are not functionally redundant and that NMT1 is the principal enzyme during embryogenesis (21). Impaired cell division may explain the failure of *NMT1^−/−^* offspring to survive and the unpredictable birth rate of the *NMT1^+/−^* offspring. This is because myristoylation regulates cell cycle progression through cytoskeletal remodeling (40). Since NMT2 was unable to compensate for NMT1 in maintaining viability and *NMT1^−/−^* offspring did not survive, we investigated the role of NMT1 in myelopoiesis (19). We observed that bone marrow-derived macrophages (BMDM) from wild-type mouse and BMDM from *NMT1^+/−^*-deficient mouse displayed different morphology when stained with Wright–Giemsa stain. BMDM of wild-type mouse had more abundant cytoplasm with cytoplasmic projections and presence of a few cytoplasmic granules than BMDM from *NMT1^+/−^*-deficient mouse. In addition, the impaired colony-forming ability of BMDM from the *NMT1^+/−^*-deficient mouse further emphasized the importance of having two functional copies of NMT1 gene in myelopoiesis. To further characterize the role of NMT1 in the differentiation process, the NMT1 activity profile was monitored during BMDM maturation. NMT1 activity in the wild-type mouse increased during the early stage of differentiation reaching a maximum at 72 h and thereafter declined. In an *in vitro* model, U937 promonocytic cell line was differentiated to monocyte/macrophage lineage, and the NMT activity profile was consistent with that of BMDM. The decline in NMT activity was due to the induction of NMT inhibitor, NIP71. To further delineate the mechanistic implication of NMT in myelopoiesis, embryonic stem cells isolated from wild-type and *NMT1^−/−^*-deficient mouse were differentiated into macrophages. The analysis of the macrophage population based on the expression of F4/80 surface marker was consistent with the biological importance of NMT1 in myelopoiesis (19).

**Figure 2:**
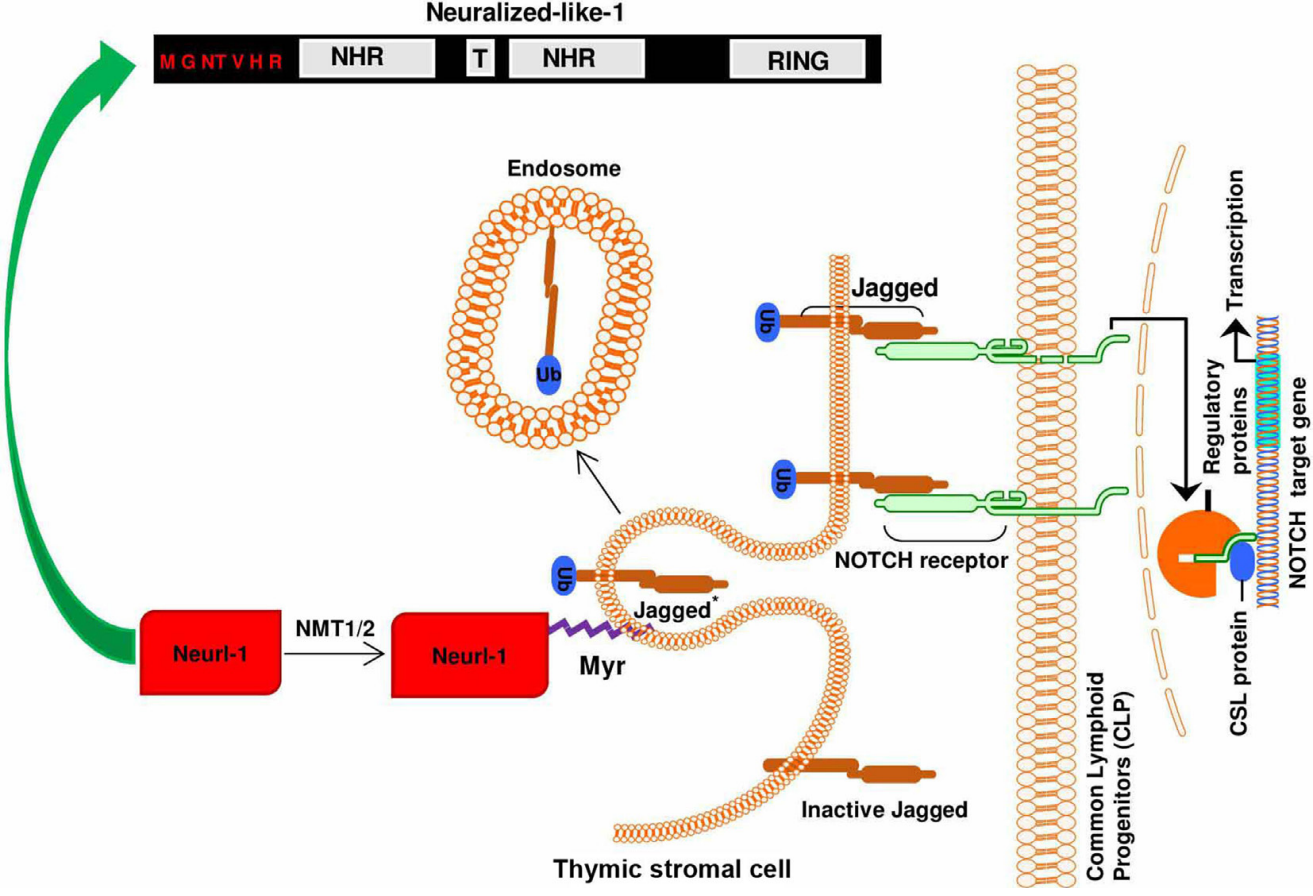
Importance of N-myristoylation of neutralized like 1 (neur1) during notch signaling: the binding of jagged 1 ligand to the notch receptor leads to the ubiquitination of functional notch ligand in the thymic stromal cell (signal-sending cell) by myristoylated neur1. The ubiquitination of jagged 1 and subsequent endocytosis and recycling mediated by myristoylated neur1 leads to regeneration of the signal/ligand (jagged 1), thus maintaining ligand–receptor interaction.

For its involvement in adaptive immunity, myristoylation is an indispensable lipid modification in the thymus during T cell development (15). Thymus is the hub for T cell development and has been shown to have high NMT activities (41). The thymic microenvironment consists of two anatomical sites: the cortex and the medulla. During the development of thymocytes, the progenitor hematopoietic precursors from bone marrow seed the cortex, while the committed immature single positive CD4 or CD8 thymocytes are found in the medulla region. Comparative analysis of the thymus in mice of NMT1^−/−^, NMT2^−/−^, or NMT1^−/−^/NMT2^−/−^ mutants showed a decrease in the medullary volume in mice of NMT1^−/−^, NMT2^−/−^; a much greater reduction in medullary volume was found in mice with double NMT1^−/−^/NMT2^−/−^ deficiency (15). It suggests that NMT1 and NMT2 play critical non-redundant roles in supporting the development of thymocytes. Medullary thymic epithelial cells regulate the intactness of medullary thymic microenvironment during T cell development. The maintenance of the thymic medullary microenvironment also requires signals from developing thymocytes (42–46). MacDonald et al. (47) and others (48, 49) reported that Notch receptor–ligand interaction is essential during early T cell lineage commitment events. To support the development of thymocytes *in vitro*, Schmitt and Zúñiga-Pflücker transduced a stromal cell line derived from bone marrow (OP9) to express the Notch ligand Delta-like-1 (50). Importantly, the Notch1 signaling pathway is regulated by myristoylated protein called neutralized like 1 (neur1). Myristoylation-mediated events target neur1 to the plasma membrane to facilitate the endocytosis, ubiquitination, and regeneration of jagged 1 in the thymus (Figure 2) (51). In agreement, the population of T cells in the spleen, lymph nodes, and thymus was significantly decreased in the NMT mutant mice when compared to the wild type. Mice deficient of NMT1 or NMT2 had a 30 and 25% reduction in thymocytes, respectively. Mice deficient of both NMT1 and NMT2 had 83% reduction of thymocytes. Nevertheless, the phenotype of T cells in mice deficient of NMT2 was similar to that of T cells from the wild-type mice. This highly suggests that NMT1 is the principal enzyme in T cell differentiation and development, which is consistent with our earlier report (19). Phenotypic analysis revealed a decreased number of Treg in the mice deficient in both NMT1 and NMT2, compared to the wild type (15).

## Myristoylation and Organization of the Immunological Synapse (IS)

Lipid rafts are microdomains of the plasma membrane that are characteristically enriched in glycosphingolipids, sphingomyelin, and cholesterol and are organized into microdomains. The observation that T-cell receptor (TCR) signaling depends in part on differential localization of signaling molecules to this microdomains raises questions about the implications of lipid rafts in T cell activation and function [for review, see Ref. (52)]. It is thought that during T cell activation lipid rafts cluster at the interface (53, 54) between T cell and antigen-presenting cell (APC) with a consequent cytoskeletal polarization (55) in responses to an antigen presented by APC. The interface between an immune cell and the APC is called IS or supramolecular activation cluster (SMAC) and it has been a subject of intense study. The formation of IS is strongly influenced by myristoylation. The IS is organized into three concentric rings—central SMAC, peripheral SMAC, and distal SMAC enclosing vital signaling proteins and excluding important inhibitory proteins. Confocal imaging showed that during formation of the IS, the central SMAC is enriched in protein kinase C (PKC), Lck, Fyn; the peripheral SMAC in adhesion molecules—LFA-1 and talin, while the distal SMAC is enriched in CD43 and CD45 (56–61). Mutation of the amino-terminal glycine residue (site of myristoylation) to alanine indicated that myristoylation is required for localization of Lck (62) and Fyn (63, 64) to the IS. Also, FAK that plays an important role in integrin mediated signal transductions forms a complex with myristoylated c-Src (65). This FAK/c-Src complex triggers downstream signaling that is vital to cell migration and adhesion depending on the subcellular localization of the complex. Migratory immune cells define their front end and rear end in an actin-dependent protrusion and retraction manner, respectively; this is evident in T cells where T cell activation leads to actin polymerization to ensure the formation of TCR signaling clusters (60, 66, 67).

**Figure 3:**
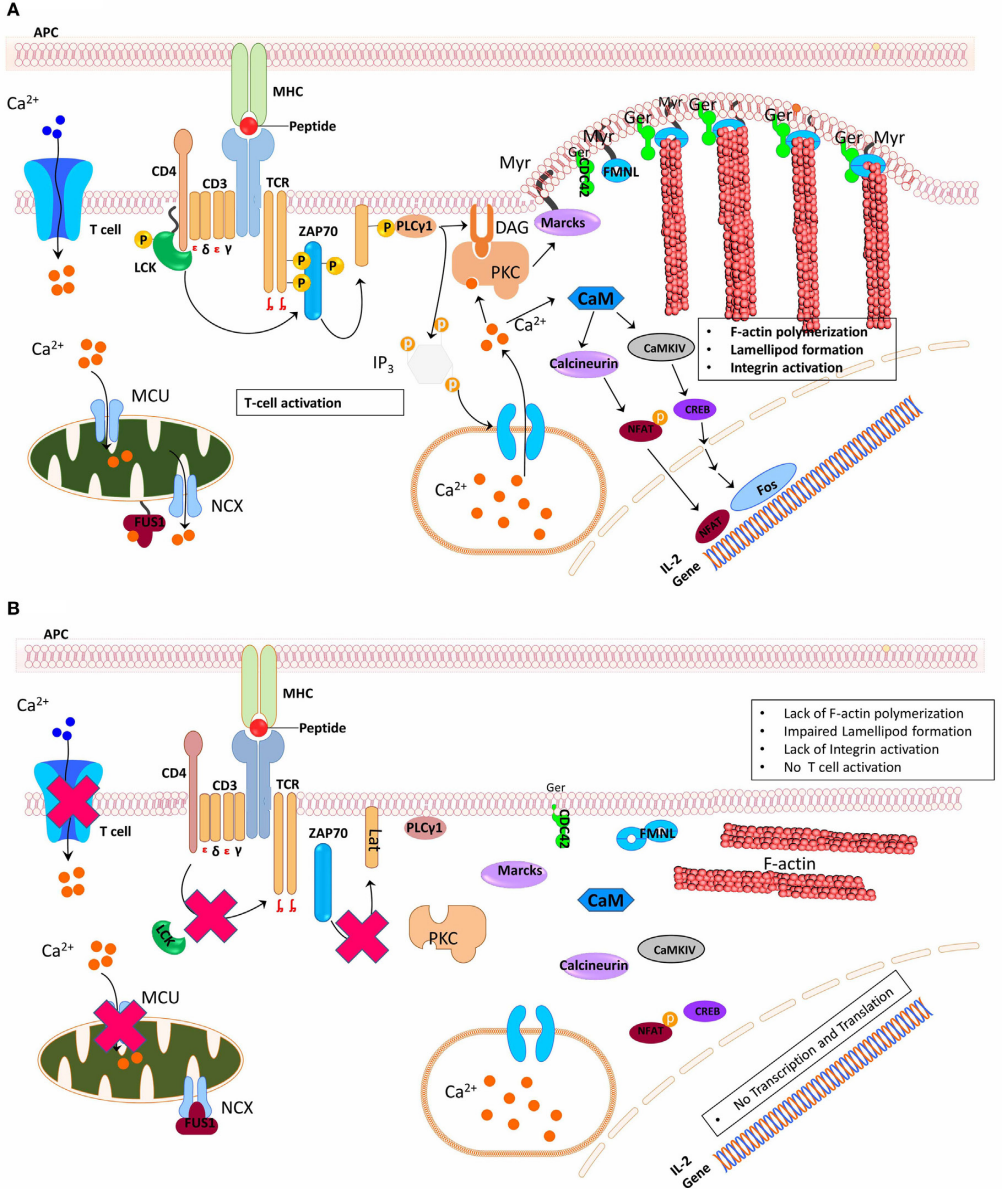
Schematic representation of N-myristoyltransferase functions during T cell activation. **(A)** Simultaneous cross-linking of T cell receptor, CD3, and CD28 on cell by the peptide presented on the major histocompatibility complex on the antigen-presenting cell (APC) induces myristoylation (Myr) of Lck which then associates with either CD4 or CD8, leading to the phosphorylation of immunoreceptor tyrosine-based activation motifs (ITAMs) of CD3. This mediates recruitment of ZAP-70 that activates Lat. Phosphorylated Lat serves as a docking site, recruiting multiple adaptor proteins essential for mediating downstream signals during T cell activation. PLC is among the several signaling proteins recruited by activated Lat, PLC mediates the activation of protein kinase C (PKC), which in turn activates myristoylated alanine-rich C kinase substrate (MARCKS). Formins (FNML) are equally activated in parallel by geranylgeranylated (Ger) cell division control protein 42, which helps in maintaining membrane plasticity that is very essential during the formation of immunological synapse. Also, inositol triphosphate, product of PLC, initiates calcium signaling by binding to its receptor on the endoplasmic reticulum. Binding of Ca^2+^ ion to FUS1, a calcium-myristoyl protein switch, promotes the release of the myristoyl group from the myristoyl-binding hydrophobic pocket and anchoring of the protein in mitochondrial membrane structures ensuring mitochondrial calcium handling. **(B)**. However, knockout or inhibition of NMT activity makes Lck to be predominantly cytosolic. Cytosolic Lck is unable to phosphorylate ITAMs and as a consequence cannot reconstitute T cell downstream signaling pathway. Similarly, non-myristoylated MARCKS and FNMLs are unable to induce membrane plasticity.

T-cell receptor signaling in T-cells is mediated through myristoylation-mediated targeting of Lck and Fyn (Src family tyrosine kinase) to the cytoplasmic domain of z chain of TCR (Figure 3A). Myristoylation of Fyn is essential for its trafficking to plasma membrane and binding with z chain of TCR (68). In addition, myristoylation-dependent localization of Lck to the CD4 receptor is very integral to the activation of T cells since un-myristoylated Lck is cytosolic and unable to facilitate TCR signaling cascades (Figure 3B) (15). In the presence of NMT activity, TCR ligation activates myristoylated Lck that in turn phosphorylates tyrosine containing immunoreceptor tyrosine-based activation motifs (ITAMs) (69). On phosphorylation of ITAMs, ZAP-70 is recruited that activates various signaling molecules leading to T cell activation (Figure 3A). According to the kinetic-segregation model of TCR triggering, in resting T cells, membrane bound tyrosine kinase Lck continuously phosphorylates the TCR/CD3 complex, but phosphorylated TCR/CD3 complexes are at the same time dephosphorylated by tyrosine phosphatases such as CD45. However, when a T cell makes contact with another cell, the IS forms spontaneously at the contact interface that leads to the exclusion of molecules with large ectodomains, such as CD45. This segregation leads to sustained phosphorylation of TCR/CD3, which recruits and activates tyrosine kinase ZAP-70 (70). Furthermore, eNOS upon being myristoylated has been shown to translocate to the IS during T cell activation, and nitric oxide was detected within the IS (71). eNOS-mediated NO production at the IS results in induced mitochondrial hyperpolarization, elevated levels of phosphorylated CD3, ZAP-70, and ERK, and increased IFN-γ production (71). Without myristoylation, eNOS would not be recruited to the IS to ensure NO production (72), which is required to mediate cGMP-dependent differentiation of naïve T cells into the Th1 phenotype *via* induction of IL12R (73). T cell polarization into either Th1 phenotype is an integral part of cell-mediated immune response and it is mediated by the translocation of microtubule-organizing center (MTOC) to the IS. MTOC positions secretory vesicles (74) in the event of cell-mediated immune response, however, the mechanism by which MTOC is translocated to the IS in not completely understood. Recently, it was shown that formins, a myristoylated protein, regulates MTOC-mediated migration to the IS *via* an actin-related protein 2/3-independent manner (75).

The role of formins in MTOC polarization in T cells (76) will not be extensively reviewed here. To date, several proteins integral to the formation of IS have been shown not only to undergo myristoylation but that their function depends on myristoylation. However, little is known about the regulation of myristoylation or NMT activity in these processes during the adaptive immune response. To have a more complete picture of the regulation of immune response, it is imperative to delineate the specific roles of NMT isozymes during immune response and exploit NMT as a potential immune modulatory target.

## Myristoylation Regulates Mitochondrial Calcium Release Activated Calcium Channels (CRAC) and is Involved in Cytotoxic T Cell Function

Electrophysiological studies conducted 20 years ago on Ca^2+^ Release Activated Ca^2+^ Channels (CRAC) on mast and T cells (77) contributed to our understanding of the relevance of the CRAC channels in mediating several biological functions. The role of CRAC channels in immune functions has been well characterized because of numerous immunodeficiencies that result from genetic defects in the structural organization and function of the CRAC. The CRAC pathway is regulated by myristoylated proteins such as CaN and Fus1 that play a crucial role in the Ca^2+^-mediated signal transduction. Ca^2+^, a second messenger, regulates a wide variety of signaling pathways and Ca^2+^-dependent protein activity that are implicated in cellular processes like cell proliferation, cell differentiation, and gene expression (78). Furthermore, in activated lymphocytes, there is a transient rise in cytosolic Ca^2+^ mediated by inositol triphosphate-dependent Ca^2+^ release from the ER. This leads to the loss of Ca^2+^ from the internal Ca^2+^ stores (ER) and the cytoplasm. The decreased level of cytosolic Ca^2+^ induces the opening and activation of the CRAC channels. Nevertheless, the gating of the CRAC channel is regulated by mitochondria. Mitochondria have been shown to buffer cytosolic Ca^2+^ level *via* uptaking and releasing of cytosolic Ca^2+^ through mitochondrial Na^+^/Ca^2+^ exchanger: a process referred to as mitochondrial Ca^2+^ handling. The protein involved in mediating mitochondrial Ca^2+^ handling is Fus1 that contains a myristoyl-Ca^2+^ switch (79). Fus1 regulates mitochondria homeostasis in both tumor and immune cells (79–82). The Fus1 null mice developed autoimmune-like syndrome with chronic inflammation and exhibited reduced IL-15 production (79, 83). In mutant CD4^+^ T cells that are deficient in Fus1, there was increased expression of Ca^2+^-regulated proteins without the consequent activation of NF-kB (79). Fus1 function in regulation of cellular Ca^2+^ level depends on myristoylation (79, 84). The binding of Ca^2+^ to myristoylated Fus1 induces the exposure of the myristoyl group from the myristoyl-binding hydrophobic pocket within Fus1. The exposed myristoyl group can then facilitate the anchoring of Fus1 to the mitochondrial membrane ensuring mitochondrial Ca^2+^ handling (Figure 3). Together, changes in protein conformation *via* the interaction of myristoyl moiety with Ca^2+^ reveal an added level of control in cellular signaling.

**Figure 4:**
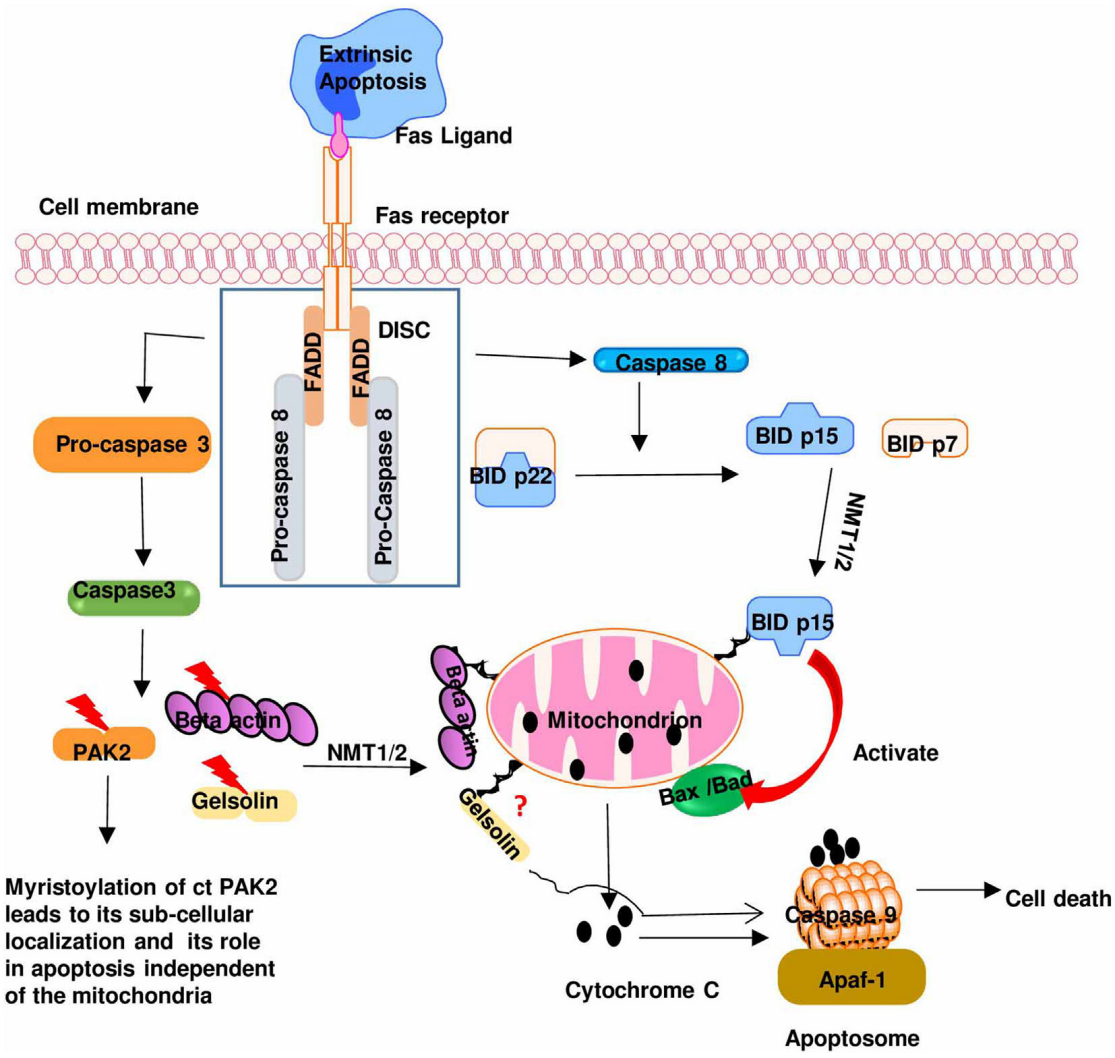
The role of myristoylation in regulating Fas/Fas ligand pathway during cytotoxic T cells activation: binding of a death ligand to Fas receptor leads to the formation of death-inducing signaling complex (DISC). DISC induces the activation of caspase 8 and caspase 3 that cleaves BID and p21-activated kinase 2 (PAK2), respectively, exposing a glycine residue that is subsequently myristoylated by NMT. This leads to subcellular localization of these pro-apoptotic proteins to the mitochondria where they mediate their pro-apoptotic effect [caspase-truncated (ct)]. Modified from Ref. (5).

In addition to its involvement in signal transduction, myristoylation also plays a role in the cytotoxic function of activated T cells *via* its role in apoptotic pathway. Cytotoxic T cells can induce apoptosis in the targeted cells through the extrinsic pathway. This pathway in part involves the ligation of Fas on the target cell membrane to the Fas ligand (FasL) present on activated cytotoxic T cells. Fas–FasL interaction results in activation of caspases in the targeted cells that induce cleavage of pro-apoptotic proteins. The posttranslational myristoylation of protein was first reported in 2000, to show that N-myristoylation of BID after its being cleaved by caspase 8 targets the myristoylated BID to mitochondrial membrane that augments the cellular pro-apoptotic activity (Figure 4) (4). As initiation of apoptosis is characterized by caspase-mediated proteolytic cleavage at the preferred glycine (85, 86), it is highly plausible that myristoylation might be the common posttranslational modification following caspase-mediated proteolytic cleavage (87). It then suggests that the posttranslational myristoylation may play a key role in cell survival and apoptosis. Heretofore, only BID and p21-activated kinase 2 are the known substrates of posttranslational myristoylation. With the *in silico* prediction analysis, coupled to an overexpression system, other substrates of posttranslational myristoylation (including but not limited to gelsolin B cell receptor-associated protein 31 and YTH domain family protein) have been discovered (4, 87, 88). These *in vitro* identified substrates were validated using a robust proteomic technique that uses new NMT inhibitors to confirm myristoylation (89). Regulation of posttranslational myristoylation during apoptosis merits further investigation with potential for drug design and development.

## Myristoylation and Immune Surveillance

Immune surveillance is the fundamental function of the immune cells, and to perform this vital biological function, immune cells must possess a remarkable ability to migrate and undergo morphological changes in response to invading pathogens. This fundamental actin-dependent process is heavily governed by dynamic cytoskeletal remodeling. The intricate relationship between immune cell polarization and reorganization of both microtubule networks and the actin cytoskeleton was demonstrated 33 years ago (90–92). Since then, several studies have revealed the importance of dynamic modulation of the cytoskeleton in immune system responses (55, 93). Furthermore, central to cytoskeletal remodeling is a group of ubiquitous eukaryotic proteins called formin-like proteins (94). Formins helps to nucleate and promote the polymerization of actin filaments. Formins have been implicated in a wide variety of actin-dependent biological processes such as wound healing, migration, vesicle trafficking, morphogenesis, cytokinesis, embryonic differentiation, and cell polarity (93, 95) as it promotes G-actin nucleation (96) and F-actin elongation (97). Formins are a diverse group of multidomain proteins characterized by their highly conserved FH2 domain that binds actin monomers and an adjacent proline rich FH1 domain that binds profilin. These domains are flanked at the N-terminal end by a conserved myristoylation consensus sequence and a number of regulatory domains such as small G protein-binding domain, that is adjacent to diaphanous-inhibitory domain and a dimerization domain, and at the C-terminal, it contains diaphanous autoregulatory domain (95, 98, 99). However, formin-like proteins, FMNL1, FMNL2, and FMNL3, make up a family of diaphanous-related formins found in mammals. FMNL1, the founding member of this family (100), is also referred to as formin-related gene in leukocytes (FRl) because it is the predominant formin in hematopoietic lineage-derived cells (spleen, lymph nodes, and bone marrow cells) (101). Furthermore, it was shown that cell adhesion, morphology, migration, and proliferation were not dysregulated in macrophages overexpressing FMNL1 containing only FH3 and FH1 but not FH2 (102). This supports the observation that FH1 domain-bound profilin, an actin-monomer binding protein, facilitates elongation of nucleated G-actin to F-actin (97), whereas FH2 domain mediates G-actin nucleation (96). It seems likely that FH1 domain elongated G-actins nucleated by actin-related proteins 2/3 (Arp2/3). Arp2/3 are well characterized nucleating factors capable of synthesizing branched actin filament (103). FMNL1 is the principal formin in regulating cell spreading and lamellipodia formation in leukocytes (104). Following this study, several studies have demonstrated the functional role of formins in modulating actin dynamics and mechanics during immune cell migration, polarization, phagocytosis, and chemotaxis (105, 106).

Importantly, the functional significance of the N-terminal myristoylation consensus sequence was investigated and found to be essential for formin’s membrane association (40). Myristoylation of a splice variant of FMNL1 localizes it to cell membrane and induces the formation of membranous bleb (107). N-Terminal myristoylation motif was not only found to be highly specific to FMNL1, FMNL2, and FMNL3 but also important for mediating actin nucleation and polymerization during lamellipodia and filopodia formation (108). Deletion of the glycine residue at position 2 demonstrated that N-terminal myristoylation of FMNL1 is required for its membrane association (107, 108). Like most myristoylated proteins, formins are cotranslationally modified by myristoylation (109), this might be a molecular mechanism for the proteins’ predominant function at the plasma membrane to ensure its activation and proper actin polymerization. The membrane-bound formins are activated by GTP-bound cell division control protein 42 (Cdc42) in response to actin polymerization signals (Figure 3A) (108, 109). Membrane association of formins is mediated by both myristoylation and electrostatic interaction, which is induced by 12 basic amino acid residues reported (108) to be next to Cdc42 binding site. Although deletion of both signals in FMNL2 rendered it cytosolic, it was observed that myristoylation alone was able to sustain its association with plasma membrane while electrostatic interaction alone was insufficient (108).

## Myristoylation and Innate Immune Response

Inflammation is considered to be the hallmark feature of the innate immune response (110). Inflammation is a protective immune response characterized by the influx of molecular mediators, neutrophils, and also monocytes that mature into inflammatory macrophages, which proliferate in response to foreign stimuli leading to the cardinal signs of acute inflammation: rubor (redness), calor (heat), tumor (swelling), and dolor (pain). Prostaglandins, a product of arachidonic metabolism, are one of the molecular mediators during acute inflammation (111). Inflammatory mediators such as TNF-α, GM-CSF, LPS, and IFN-γ stimulate arachidonic metabolism in macrophages. LPS have been shown to prime macrophages for enhanced arachidonic metabolism (112–114). During LPS-mediated priming, a subset of macrophage proteins with molecular weight of 40, 42, and 68 kDa has been shown to be myristoylated during inflammatory and immune responses in macrophages (115–117). The myristoylation of the 48-kDa macrophage protein was reported to be expressed in macrophages that were activated *in vivo* by intraperitoneal administration of inactivated *Cryptosporidium parvum* (118) and *in vitro* activated with physiological concentration of IFN-γ (118). However, IFN-α or -β was not effective at inducing myristoylation of this macrophage protein. The biological functions of this macrophage protein were unknown at the time, but it is now known as the effector substrates of PKC. This myristoylated macrophage protein is MARCKS (119). Myristoylation of MARCKS is a required prerequisite for its effective colocalization with PKC (113) and critical for mediating the inflammatory signaling. TNF-α also primes neutrophils for enhanced PKC-dependent events by inducing the transcription, translation, and myristoylation of MARCKS. LPS induces MARCKS expression in murine macrophages and neutrophils (120). Furthermore, MARCKS represents 90% of all proteins induced in response to TNF-α in human neutrophils (112). This suggests that myristoylation-mediated events are important in TNF-α-dependent signal transduction. This is consistent with the observation that primed neutrophils when exposed to a second chemotactic peptide lead to increased levels of phosphorylated MARCKS unlike unprimed neutrophils (112). Following MARCKS discovery in 1989, the MARCKS protein has continued to be the subject of active investigation especially for its role in cell motility and phagocytosis (115). Membrane localization of MARCKS is mediated by two signals: N-terminal myristoylation, which is essential for stable membrane association, shown in mutational studies and the electrostatic interaction between the basic residues in the effector domain of MARCKS and acidic lipids in the plasma membrane. Phosphorylation of MARCKS introduces negative charges in the effector region leading to charge repulsion and translocation to the cytosol. It has been proposed that dephosphorylated MARCKS preferentially associates with the plasma membrane and is able to cross-link actin filament forming a rigid meshwork. However, upon phosphorylation, MARCKS becomes predominantly cytosolic where it remains bound to actin but cannot cross-link actin. Cytosolic MARCKS still retains its myristic acid group and is able to relocate to the plasma membrane when it is dephosphorylated by a phosphatase (121). Thus, the cycle of plasma membrane attachment and detachment of MARCKS controls the cell’s phenotypic plasticity by regulating the ability of MARCKS to cross-link actin (2, 115, 122, 123).

Upon binding to LPS, TLR4 dimerizes and triggers a cascade of intracellular signaling, which help to recruits all four TIR domains containing adaptor molecules [for review, see Ref. (124, 125)]. TIR domain containing adaptor protein, TRAM, colocalizes with TLR4 in the plasma membrane and Golgi apparatus where it mediates TLR4 signal transduction. Again, the membrane association and localization of TRAM depend on the N-myristoylation at a putative myristoylation site that rendered TRAM cytosolic (18). This is shown in a mutagenesis study that when the N-terminal glycine residue of TRAM protein was mutated to alanine, TRAM failed to mediate LPS-elicited immune response or elicit IFN regulatory factor 3 and NF*k*B signaling (18). Thus, TRAM is regulated by myristoylation and this cotranslational modification enables TRAM to colocalize with PKC, which mediates phosphorylation of TRAM in response to LPS stimulation (18). A recent study reported a hitherto unappreciated role of TRAM in TLR7-mediated IRF3 activation (126). Furthermore, impaired myristoylation of TRAM cannot reconstitute TLR7-mediated activation of CCL5, IFN-α, and IFN-β reporter gene activity (126). Taken together, these clearly demonstrate that myristoylation plays an important role in innate response *via* its property in mediating the controlled localization of MARCKS and TRAM to cell membrane to interact with other innate mediators during signal transduction.

## Myristoylation and eNOS Regulation

The pleiotropic characteristic of NO has been the subject of active investigation after it was reported to be produced by activated macrophages three decades ago. Though inducible nitric oxide synthase (iNOS) accounts for most NO from macrophages, constitutive nitric oxide synthase from other immune cells produces considerable amount of NO. The pleiotropic roles of NO in regulating innate and adaptive arm of the immune system have been extensively reviewed (127–129). NO is a ubiquitous second messenger generated by oxidative reduction of l-arginine by a family of isozymes called nitric oxide synthase. This group of isozymes (eNOS, iNOS, and neuronal nitric oxide synthase) shares a common homology in the primary amino acid sequence at the C-terminal region; however, there is a significant diversity at the N-terminal region. These sequence divergences at the N-terminal region have been speculated to be responsible for the subcellular distribution of NOS (72). The N-terminal region of eNOS contains myristoylation consensus site and palmitoylation motif, which are evidently absent in other isoforms (130). eNOS is the only NOS that localizes to the plasma membrane. Like many myristoylated proteins, membrane association is indispensable to its biological function, without which eNOS becomes predominantly cytosolic with impaired enzymatic activity (130). A separate report demonstrated that N-myristoylation of eNOS was a prerequisite for the enzyme to undergo palmitoylation and it was restricted to the membrane-bound eNOS (131, 132). Many myristoylated proteins require additional form of lipid modification or electrostatic interaction to stabilize the protein. It is possible that palmitoylation helps in stabilizing the membrane-bound eNOS. Myristoylation directly regulates the biological activity of eNOS by localizing eNOS to the plasma membrane specifically in the caveolae where caveolin-1 binds to eNOS preventing the binding of CaM, when calcium levels are low, thus rendering the enzyme inactive (133, 134). However, increased calcium fluxes activate calcium/CaM complex, which displaces caveolin-1, leading to interaction of eNOS and calcium/CaM complex and rendering eNOS active (7). Accumulating evidence points to the fact that membrane localization of eNOS is important for efficient diffusion of NO to the extracellular spaces (7, 71, 130, 131, 135–137).

## Role of NMT in the Pathogenesis of HIV/AIDS

**Figure 5:**
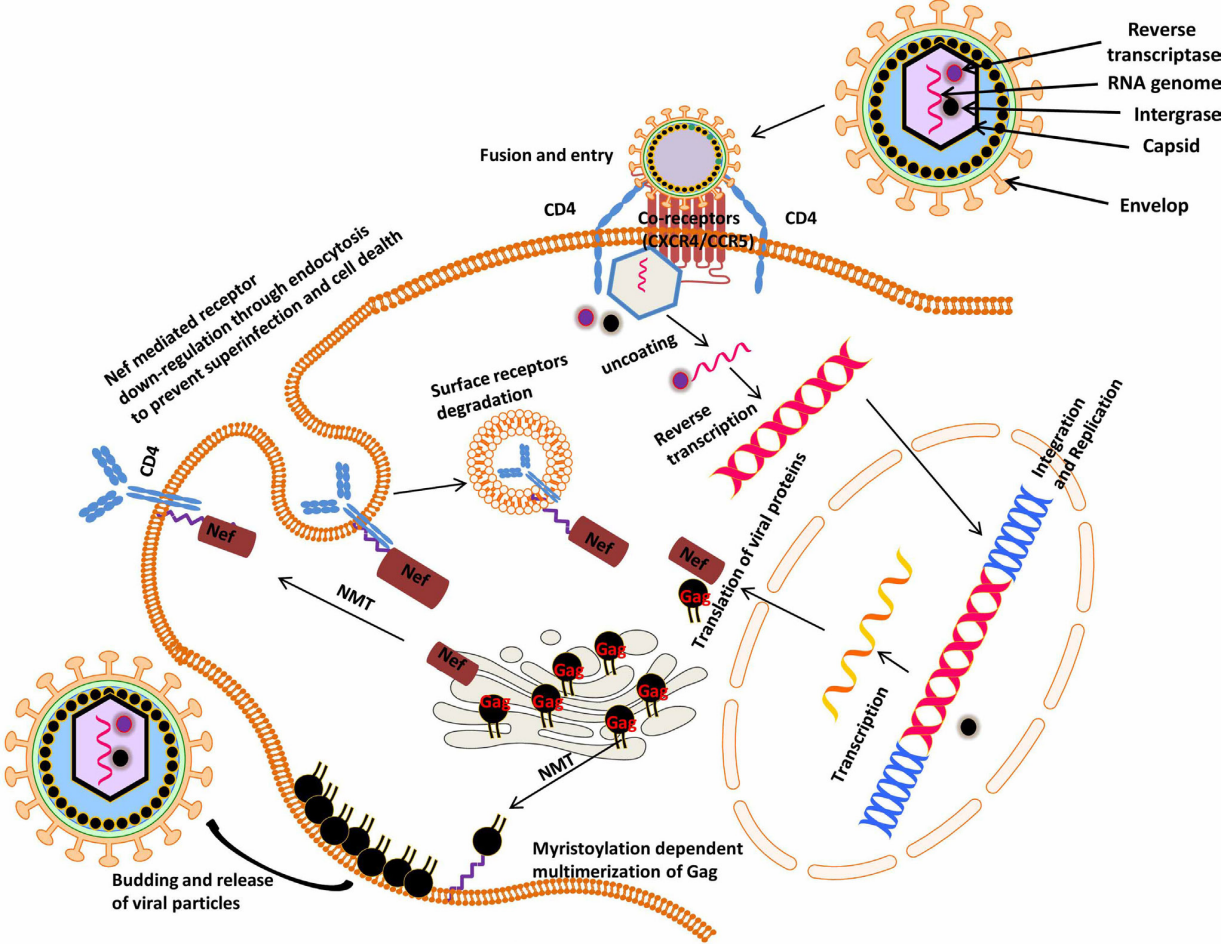
Myristoylation-dependent regulation of human immunodeficiency virus (HIV) viral proteins: the principal targets of HIV are mostly cells that express CD4 and CCR5 or CXCR4 surface receptors vis-à-vis CD4 T lymphocytes, macrophages, monocytes, and dendritic cells. Upon entering its target cells, HIV integrates inside its host genome and thus hijacking the host gene expression machinery. Subsequently, there is production of both host and viral proteins that drive the progression of HIV to AIDS. For example, the viral protein, Nef, is cotranslationally modified by the host’s NMT, which ensures its interaction with and downregulation of the host cell surface receptors (CD4 and MHC1). On the other hand, NMT mediates the assembly and multimerization of Gag at the plasma membrane during budding and viral release events.

Human immunodeficiency virus is an obligate intracellular parasite that uses the machinery of the host cell to undergo replication. The genomic organization of HIV shows that it contains three major genes, gag, pol, and env, which are flanked by long terminal repeat sequence at each end of the genome, which it uses to insert itself in the host genome (12, 138). These genes encode structural proteins and enzymes needed for replication, HIV 1 also encodes two regulatory genes, tat and rev, and four accessory genes, vif, vpu, vpr, and nef, which are required for the establishment and maintenance of viral infectivity. Over the years, studies on HIV-Nef protein have revealed that it plays important roles in HIV pathogenesis (139, 140). There are also reports indicating that HIV-Nef has a negative influence on viral growth (141, 142). During viral life cycle, Nef is expressed at higher levels and is involved in downregulation of CD4^+^ receptors from the cell surface thus inhibiting coinfection and consequently maintaining a high viral load *in vivo* (Figure 5) (25, 143). Studies on individual infected with an attenuated HIV strain containing a non-functional Nef demonstrated that progression to AIDS took at least a decade, thus calling “Nef” a negative factor is a misnomer (144, 145). Myristoylation of Nef is essential for its activity (146, 147), and deletion of N-terminal myristoylation site on Nef crippled its interaction with the cytoplasmic tail of CD4 (148, 149). NMT1 isoform has been shown to preferentially associate with HIV Nef (150). These studies suggest that downregulation of NMT would delay HIV infection since myristoylation anchors Nef to the plasma membrane, where it associates with surface receptors marking them for degradation. Although this study has been instrumental in implicating the role if NMT in HIV-infection and an important step in elucidating the substrate specificity inherent in NMT isozymes during HIV infection, there are two limitations to this study. The study was carried out in an overexpression system and their observation was not made in principal HIV target cells or in the presence of productive HIV infection. Therefore, NMT1/NMT2 catalysis during productive HIV infection in its principal target cells (i.e., CD4 T cells) should be explored to better understand its role in HIV infection and then as a potential therapeutic target. Gag is among the structural proteins expressed in late HIV life cycle that mediates recruitment and budding of newly synthesized HIV virions (151, 152). Gag is targeted to the plasma membrane by NMT (152–154), allowing protein–protein and protein–lipid interactions. Consequently, this interaction allows the recruitment of Gag molecules, RNA, and various viral proteins to the plasma membrane and hence budding of newly synthesized virion (155) (Figure 5). HIV particles in which Gag’s amino-terminal glycine was mutated to alanine were unable to produce viral particles, and supernatant from these transfected cells failed to infect CEM cells. This suggests that non-myristoylated Gag mutants cannot bind tightly to the plasma membrane and as such are unable to be assembled into active viral particles (156). Furthermore it has been reported that NMT2 preferentially associates with Gag (150). Based on these findings, NMT is considered a potential drug target for the inhibition of retroviral assembly (157), although this novel strategy will equally impair normal host cells’ NMT-mediated processes. Our earlier reports together with the studies from other group have demonstrated that NMT1 isoform is the indispensable NMT during normal biological activities (15, 19, 21). Hence, to improve the efficacy of NMT inhibitors there is a need to further delineate the specific roles of NMT isoforms during HIV infection. There are several reports in the literature which confirm that NMT is crucial in the viral pathogenicity and progression to AIDS (146, 149, 152–154, 156). High expression of NMT in HIV-infected cells will be expected since HIV has no NMT protein; instead, it uses the host NMT to ensure myristoylation of its virulent factors. Counterintuitively, lower levels of NMT were found after a concomitant increase in virulent factors during HIV infection (158). In the same study, it was shown that NMT inhibitors were cytotoxic to HIV-infected cells. This shows that HIV infection relies on recruitment of host cell NMT, since the viral proteins p17gag and Nef require myristoylation to confer infectivity (19, 68, 158, 159). It would be insightful to perform a time kinetic study to shed light on the enzymology of NMT during productive HIV infection. It is plausible that one of the reasons for the lower expression of NMT in HIV1-infected CEM cell lines is the viral strategy to lower the levels of membrane-bound Lck and other myristoylated proteins that are important for the normal functioning of the cell so as to maintain persistent viral infection. Studies are underway in our laboratory to delineate the specific NMT enzymology during HIV infection.

The decreased levels of NMT may be responsible for the impaired immune response in HIV-infected individuals as well, since myristoylated proteins (members of the non-receptor family kinases) are essential for antiviral responses and normal T cell function (as discussed above). Moreover, changes in T cell function are associated with HIV infection due to the chronic activation of the immune system. It has been reported that there is general decline in TCR and CRAC pathway activation in PBMC of HIV1-infected individuals (160). When a PhosFlow analysis was performed on stimulated PBMC, it was found that the phosphorylation of proteins in TCR signaling was blunted in HIV1-infected compared to control subjects (160). Most of what is known about human immunology comes from HIV/AIDS-related studies and it is not surprising, therefore, that the functional implication of myristoylation-mediated immune events has only been studied in HIV infection study. In the future, there is an urgent need to characterize the specific functions of NMT isozymes in different immune deficiency states and different autoimmune diseases. This will undoubtedly lead to the better understanding of the biology and enzymology of NMT during immune function and regulation.

## Conclusion

Protein N-myristoylation has been shown to be an important evolutionarily conserved modification of proteins implicated in different physiological processes like cell proliferation, differentiation, survival, and cell death. With the advent of better study models, the intricate role of NMT in mediating both innate and adaptive immune responses will continue to be a rewarding area of research in immunology.

## Author Contributions

DU, R-CS, SG, TB, SVS, and AS together wrote the manuscript. AS directed the overall study.

## Conflict of Interest Statement

The authors declare that the research was conducted in the absence of any commercial or financial relationships that could be construed as a potential conflict of interest. SVS served as a Scientific Advisor to VastCon Inc. She has helped in the organization of the manuscript and contributed towards writing of the manuscript. All other authors declare no competing interests.
